# Padi6 expression patterns in buffalo oocytes and preimplantation embryos

**DOI:** 10.1590/1984-3143-AR2023-0146

**Published:** 2024-03-22

**Authors:** Qinqiang Sun, Yuan Yang, Yue Zhang, Dongrong Chen, Haiying Zheng, Guangsheng Qin, Qiang Fu

**Affiliations:** 1 College of Animal Science and Technology, Guangxi University, Nanning, Guangxi, China; 2 Buffalo Research Institute, Chinese Academy of Agricultural Sciences, Nanning, Guangxi, China

**Keywords:** maternal-effect genes, Padi6, oocyte, preimplantation embryo, immunofluorescence

## Abstract

The subcortical maternal complex, which consists of maternal-effect genes, plays a crucial role in the development of oocytes and preimplantation embryo until the activation of the zygote genome. One such gene, known as peptidyl-arginine deiminase VI (*Padi6*), is involved in the oocyte maturation, fertilization and embryonic development. However, the precise function of *Padi6* gene in buffalo is still unclear and requires further investigation. In this study, the sequence, mRNA and protein expression patterns of *Padi6* gene were analyzed in oocytes, preimplantation embryos and somatic tissues of buffalo. The coding sequence of gene was successfully cloned and characterized. Real-time quantitative PCR results indicated an absence of *Padi6* transcripts in somatic tissues. Notably, the expression levels of *Padi6* in oocytes showed an increased from the germinal vesicle stage to metaphase II stage, followed by a rapid decrease during the morula and blastocyst stages. Immunofluorescence analysis confirmed these findings, revealing a noticeable decline in protein expression levels. Our research provides the initial comprehensive expression profile of *Padi6* in buffalo oocytes and preimplantation embryos, serving as a solid foundation for further investigations into the functionality of maternal-effect genes in buffalo.

## Introduction

Buffalo species hold significant economic and biological importance, especially in tropical and subtropical regions. Buffaloes serve as a multipurpose animal, providing draft power, milk, and meat. However, their reproductive efficiency remains lower compared to other domestic animals, posing a challenge to the advancement of dairy buffalo industry. Consequently, comprehensive research on buffalo oocytes and embryos is imperative for improving the reproductive capabilities. The cloning and identification of crucial genes related to oocyte and embryo development are pivotal in elucidating the underlying factors contributing to the lower reproduction performance, and promoting the progress of buffalo industry.

Maternal effect genes (MEGs) play a crucial role in oocyte maturation and the development of embryo. Following fertilization, the zygotic genome remains inactive, relying on MEGs to provide the encode mRNAs and proteins for processes such as cell division, epigenetic reprogramming, chromatin remodeling, and zygote activation cascades (Jukam et al., 2017). The subcortical maternal complex (SCMC), which is present in oocytes and preimplantation embryos, consists of multiplex protein encoded by MEGs. While SCMCs have minimal impact on oocyte maturation and fertilization, they are essential for early cleavage and the reprogramming of preimplantation embryos (Lu et al., 2017; Zhang and Smith, 2015).

SCMCs, which are functionally conserved in various mammalian species, consists of eight proteins: oocyte-expressed protein (OOEP), peptidyl-arginine deiminase VI (PADI6), zinc finger BED domain-containing protein 3 (ZBED3), transducin-like enhancer protein 6 (TLE6), KH domain-containing protein 3 (FILIA), and NOD-like receptor family pyrin domain containing 2 (NLRP2), NLRP5, and NLRP7 (Zhu et al., 2015; Mahadevan et al., 2017; Monk et al., 2017; Xu et al., 2016; Zhang et al., 2021). *Padi6* encodes an enzymes belonging to the peptidyl-arginine deiminase family, which converts arginine residues to citrulline (Witalison et al., 2015). Previous studies conducted in mice and humans have demonstrated that*PADI6*is highly expressed in oocytes and preimplantation embryos, where it colocalizes with other components of the SCMC (Yurttas et al., 2008; Yu et al., 2014). PADI6 is necessary for the formation of the oocyte lattices, which are believed to serve as ribosomal storage for preimplantation embryo (Yurttas et al., 2008). The absence of Padi6 in embryos leads to arrest at the 2-cell stage and impaired activation of the embryonic genome, indicating its significance as a novel MEGs (Esposito et al., 2007). In humans, mutations in PADI6 have been linked to female infertility (Qian et al., 2018). Liu et al. reported PADI6 plays a critical role in the formation of oocyte cytoplasmic lattices in mammals. However, little is known regarding the expression patterns of *padi6* genes during oocytes and embryos development in buffalo species. This study aims to analyze the gene expression profiles of *Padi6* to elucidate the roles of the MEGs during embryonic development. Our findings have significant implications for improving *in vitro* maturation systems and addressing infertility issues in buffalo.

## Material and methods

The Animal Experimentation Ethics Committee of Guangxi University (Nanning, China) provided guidance and approval for the experiments conducted in this study.

### Collection, maturation and fertilization of oocyte

Buffalo ovaries were procured from a slaughterhouse and promptly transported to the laboratory at 37°C. Oocytes were aspirated from follicles with a diameter of 2–8 mm, and subsequently washed with TCM-199 medium supplemented with 20 mM HEPEs, 5 mM sodium bicarbonate, and 0.06 mg/mL penicillin. Intact cumulus oocyte complexes were selected for *in vitro* maturation, following a previously published protocol (Huang et al., 2018). Following maturation, the cumulus oocyte complexes were washed, and the cumulus cells were gently removed by pipetting. For *in vitro* fertilization, thawed buffalo semen was adjusted to a concentration of 2 × 10^6^/mL, and 15 μL of spermatozoa were added to each fertilization drop containing oocytes. The fertilization drops were then incubated at 38.5°C in a humidified incubator with 5% CO_2_ for a duration of 24 h.

### In vitro cultivation of embryos

After fertilization, presumptive zygotes were separated from the fertilization drops, and they underwent three washes with a modified Tyrode’s In vitro cultivation medium consisted of 36% TCM-199, 10% fetal bovine serum, 0.06 mg/mL penicillin, and 0.1mg/mL streptomycin. Subsequently, the zygotes were transferred to 100-μL culture drops. Every 48 h, half of the original IVC medium in each culture drop was replaced with a similar volume of fresh medium. The zygotes were cultured for a period of 7–8 days in a humidified incubator with 5% CO_2_ at 38.5°C.

### Reverse transcription and quantitative real-time PCR (qRT-PCR)

Total RNA was extracted from oocytes, preimplantation embryos (2-cell, 4-cell, 8-cell, morula, and blastocyst), and various tissues (heart, liver, spleen, lung, kidney, brain, testicle, ovary, and others) using the RNAiso Plus Kit (Takara, Kusatsu, Japan) following the manufacturer’s instructions. To ensure adequate RNA yield, 200-300 oocytes, 100-150 2-cells embryos, 50-80 4-cells embryos, 20-40 8-cells embryos, approximately 20 morulae and 10 blastocysts were used for extraction. The concentration of RNA in each sample was quantified using a NanoDrop 1000 spectrophotometer (Thermo scientific, MA, USA). The isolated RNA was eluted in RNase-free water and promptly subjected to reverse transcription PCR. First-strand cDNA synthesis was performed using the PrimerScript RT Master Mix (Takara), comprising PrimeScript RT, EnzymeMix I, PrimeScript buffer, and RNase-free water in a final volume of 10 μL. The reaction tubes were incubated at 37°C for 15 min, followed by 85°C for 5 s to inactivate the reaction. qRT-PCR analysis was performed using a SYBR-premix Ex Taq Kit (Takara) and a LightCycler 480 instrument (Roche, Basel, Switzerland), as described in previous studies (Huang et al., 2018; Fu et al., 2016). The primer sequences are detailed in Supplementary Table S1. Gene expression levels were determined using the 2^−ΔΔCT^ method. *GAPDH* expression serving as the control group.

### Semi-quantitative analysis of Padi6 transcript in somatic tissues

To evaluate the expression of*Padi6* in somatic tissues, semi-quantitative PCR experiments were conducted. First-strand cDNA synthesis was carried out as detailed in section 2.3. PCR amplification was performed with the following cycling conditions: initial denaturation at 95°C for 5 min; followed by 34 cycles of denaturation at 95°C for 15 s, annealing at 60°C for 15 s, and extension at 72°C for 30 s. A final extension step was conducted at 72°C for 5 min. The expression of *GAPDH* was used as a positive control. Subsequently, the PCR products were subjected to analysis by gel electrophoresis on 2% agarose gels stained with SYBR.

### Immunofluorescence analysis

Buffalo oocytes and preimplantation embryos (10 cells in each group) were underwent three washes with PBS. Following this, the oocytes and embryos were fixed at room temperature for 30 min using 4% paraformaldehyde and permeabilized with 0.1% Triton X-100 for 10 min. Subsequently, oocytes and embryos were incubated in a blocking solution containing 5% goat serum for 1 h at room temperature, followed by incubation with diluted (1:50) PADI6 primary antibody (Cwbio, Beijing, China) overnight at 4°C. Then the oocytes and embryos were then incubated with a FITC-conjugated secondary antibody (Cwbio, Beijing, China) in the dark at room temperature for 1 h. Fluorescence images were captured using a fluorescence microscope (Zeiss Z2, Oberkochen, Germany).

### Statistical analysis

Quantitative data were presented as the mean ± standard error of the mean from a minimum of three independent experiments. These data were then analyzed using one-way ANOVA for evaluation. Statistical significance was considered when *P* ≤ 0.05.

## Results

### Cloning and characterization of *Padi6* gene

**Figure 1:**
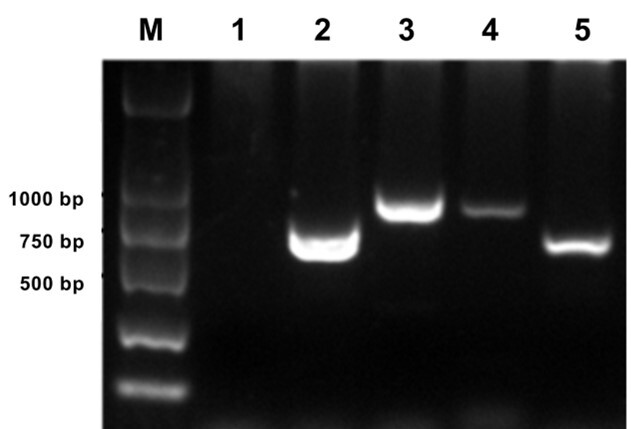
Electrophoresis results of PCR amplification products. M: DNA Marker; 1: blank control; 2: 576-bp fragment; 3: 712-bp fragment; 4: 639-bp fragment; 5: 508-bp fragment.

The *Padi6* gene from buffalo was successfully cloned into four fragments of 576 bp, 712 bp, 639 bp, and 778 bp (Figure 1). Subsequently, these fragments were assembled to generate a 2121-bp sequence, encompassing a region of the partial coding sequence. The open reading frame (ORF) of the gene encodes a protein consisting of 765 amino acids, with leucine, serine, and glutamine accounting for 10.3%, 8.4% and 8.2% of the amino acid composition, respectively. The predicted translation product exhibits a molecular mass of 85.35 kD and an isoelectric point of 6.50. Furthermore, the PADI6 protein contains three distinct domains. The ProtScale software (Gasteiger et al. 2005) calculated a grand average of hydropathicity of 0.188, indicating the hydrophilic nature of the PADI6 protein.

**Figure 2:**
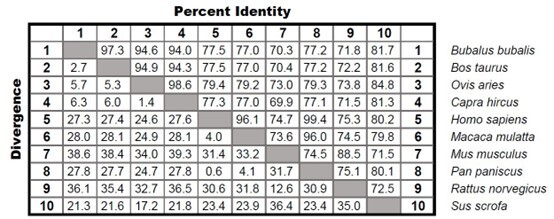
Sequence homology analysis of different mammalian species.

To assess the homology of the buffalo *Padi6* gene, the BLAST tool was utilized to compare it with the *Padi6* sequences from various mammalian species, such as sheep (XM_027965660.1), goat (XM_018054709.1), cattle (XM_002685797.5), boar (XM_013999025.2), rat (NM_001191766.1) and mouse (NM_153106.2), macaque (XM_001089244.4), orangutan (XM_003814444.1), and human (NM_207421.4). The BLAST analysis revealed a high homology between the buffalo *Padi6* gene and cattle (97.3%), goat (94.6%) and sheep (94.0%) (Figure 2A). Conversely, the lowest homology was observed with the mouse sequence (70.3%), suggesting the evolutionary conservation of the *Padi6* gene among diverse mammalian species.

### Tissue distribution of *Padi6* gene

**Figure 3:**
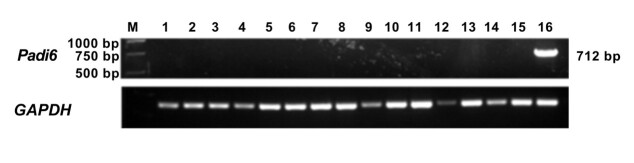
*Padi6* mRNA expression profile based on qRT-PCR of multiple tissues. 1: heart, 2: liver, 3: spleen, 4: lung, 5: kidney, 6: brain, 7: spinal cord, 8: tongue, 9: throat, 10: stomach, 11: muscle, 12: fat, 13: testicle, 14: uterus, 15: ovary, 16: oocyte.

To investigate the distribution of *Padi6* mRNA in different tissues, a semi-quantitative analysis was performed using qRT-PCR. Various somatic tissues including the heart, liver, spleen, lung, kidney, brain, spinal cord, tongue, throat, stomach, muscle, fat, and uterus, as well as germ line tissues such as testicle and ovary, were examined. The expression levels of *Padi6*mRNA were normalized to the expression of *GAPDH*, which functioned as an internal control for reverse transcription. Notably, distinct and specific expression of *Padi6* was exclusively observed in oocytes, with no detectable transcripts in other tissues (Figure 3). This highly specific expression pattern suggests a specialized role for *Padi6* in oocyte function.

### *Padi6* expression patterns in oocytes and preimplantation embryos

**Figure 4:**
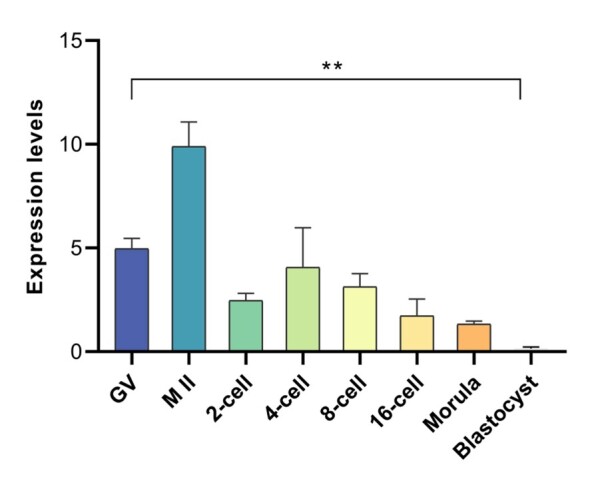
*Padi6* mRNA expression levels in buffalo oocytes and preimplantation embryos. **Compared with control group, *P*<0.01.

Upon further analysis using qRT-PCR, the presence of *Padi6* mRNA was confirmed in the germinal vesicle (GV) and metaphase II (MII) stage embryos, as well as during preimplantation embryos development, encompassing the 2-cell, 4-cell, 8-cell, 16-cell, morula, and blastocyst stage. The expression of *Padi6* mRNA exhibited a significant increase from the GV to the MII stages, with the highest level of expression observed at the MII stage. Subsequently, a gradual decrease in expression was noted during preimplantation embryo development, although a slight increase was observed at the 4-cell stage (Figure 4). These findings suggest that *Padi6* is predominantly expressed prior to the MII stage and plays important roles in oocyte maturation.

### PADI6 proteins expression and subcellular localization

**Figure 5:**
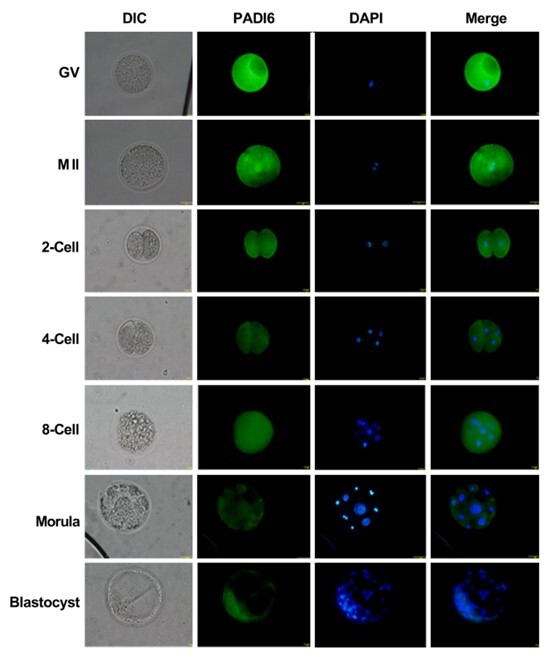
Immunofluorescence of PADI6 protein in buffalo oocytes and preimplantation embryos.

To further investigate the expression and subcellular localization of the PADI6 protein, immunofluorescence analysis was conducted. The results revealed the presence of PADI6 protein in buffalo oocytes and preimplantation embryos. Notably, the fluorescence intensity of GV stage oocytes was higher in comparison to oocyte at the MII stage. In both GV and MII stage oocytes, the PADI6 protein was predominantly localized in the cytoplasmic region (Figure 5). Furthermore, zygotes exhibited a significant decrease in PADI6 expression compared to MII stage oocytes. Throughout the developmental stages from 2-cell to 8-cell embryos, similar fluorescence signals were observed, which gradually decreased from the morula stage onward. At the blastocyst stage, PADI6 protein was expressed at extremely low levels in the cortical region. These results are consistent with the mRNA expression pattern and suggest that PADI6 protein primarily functions during oocyte maturation and preimplantation embryonic development from 2–8 cell divisions.

## Discussion

Female gametes are transcriptionally inactive during fertilization. The activation of the zygote genome occurs at different stages of embryonic development in various species. In mice, it happens during the later one-cell to early 2-cell stage, while in pigs, it occurs at the 4-cell stage. Primates experience genome activation between the 4-cell to 8-cell stage, and cattle between the 8-cell to 16-cell stage (Pisani et al., 2010). The regulation of early zygotic development relies on pre-existing factors encoded by MEGs (Evsikov and Marín de Evsikova, 2009). Among these factors, the products of *Padi6* is one of the earliest factors to influence embryogenesis. During oogenesis, PADI6 functions by catalyzing the deamination of arginine residues in proteins, potentially contributing to cytoskeletal reorganization in oocytes and preimplantation embryos. Studies on *Padi6*-deficient mice have shown that these animals are infertile due to developmental defects in preimplantation embryos, highlighting the crucial role of PADI6 in female fertility (Rezaei et al., 2021). Furthermore, previous research has linked PADI6 to other reproductive deficiencies, strengthening the association between PADI6 and infertility, miscarriages, and molar pregnancies (Qian et al., 2018; Maddirevula et al., 2017). At the cellular level, PADI6 is the first identified oocyte-specific protein that localizes to cytoplasmic lattices in mice. The cytoskeletal reorganization mediated by PADI6 is critical for regulating organelle positioning and redistribution (Esposito et al., 2007).

The expression patterns and functions of *Padi6* in buffalo have not been previously characterized. This study aimed to clone and analyze the gene for the first time, providing a foundation for further validation of its function. The coding sequence of buffalo *Padi6* is a 2.2 kb sequence and consists of 17 exons, similar in length to bovine, mouse, swine, and macaque. However, the number of exons varies among different animal species, indicating distinct gene organization in buffalo *Padi6*. The ORF of buffalo *Padi6* encodes a predicted protein of 765 amino acids with a molecular weight of 85.35 kD, which is shorter than the corresponding protein in mouse and swine.

Previous studies have described the expression profile of *Padi6* transcripts (Esposito et al., 2007; Chavanas et al., 2004). In our study, *Padi6* transcripts were not detected in somatic tissues, suggesting a tissue-distribution pattern similar to that of other domestic animal species. During oocyte maturation, until embryo cleavage, *Padi6* mRNA exhibited a rapidly decrease. Interestingly, while previous studies reported low expression levels in morula and blastocysts (Pennetier et al., 2006). We found abundant PADI6 protein in buffalo oocytes and preimplantation embryos, suggesting a potential persistent of *Padi6* in buffalo embryo development.

MEGs play a crucial role in various stages of reproduction, including oogenesis, oocyte maturation, and preimplantation embryo development. Buffalo *Padi6* gene exhibits a distinct expression pattern during the development of oocytes and embryos. The mRNA molecules are highly transcribed and accumulated in the cytoplasm of oocytes during oogenesis, but degrade rapidly after fertilization. Therefore, it is essential to investigate the regulatory mechanisms underlying oocyte and preimplantation embryo development in order to gain a comprehensive understanding of these processes. Furthermore, such investigations have important implications for advancements in *in vitro* embryo culture, somatic cell cloning, and transgenic breeding.

## Conclusion

In conclusion, this study provides the first characterization of *Padi6,* a component of the SCMC, during oocyte and preimplantation embryo development in buffalo. The mRNA transcript and protein expression profiles suggest that *Padi6* may function in buffalo oocyte maturation and preimplantation embryo cleavage. These findings contribute to our understanding of the fundamental biological processes in SCMCs during buffalo embryo development.

## Supplementary Material

Supplementary material accompanies this paper.

Supplementary Table S1

This material is available as part of the online article from https://doi.org/10.1590/1984-3143-AR2023-0146
