# Supplementary material for: Padi6 expression patterns in buffalo oocytes and preimplantation embryos
Source: Anim Reprod. 2024 Mar 22;21(1):e20230146. doi: 10.1590/1984-3143-AR2023-0146 (PMC10984561; doi:10.1590/1984-3143-AR2023-0146)
Supplement: Supplementary Table S1 [file 1984-3143-ar-21-1-e20230146-suppl01.pdf]

Supplementary Table S1			
gene	primer name	primer sequence	PCR production
PADI6	PADI6-1F	GGTTGTCAGAAGTTACCGTG	411
	PADI6-1R	GGAGAGCTCAATGCCTGTGA	
	PADI6-2F	ATGTGCGAGTCCTTCTCCGT	576
	PADI6-2R	CCAGAACCAACTCGAAGGTC	
	PADI6-3F	CTCCACACCTCCAAGGAAGA	712
	PADI6-3R	AAGCTGCTGCCAATAAGGAC	
	PADI6-4F	GCCAGCATAGATGTCATTGG	639
	PADI6-4R	CCATAATGACCATCTCCAGC	
	PADI6-5F	GAGCAGACCAAGAAGCTCTA	508
	PADI6-5R	CAGAACAGCTAGGACTTTAC	
